# Stage-Specific Impacts of Climate Change on Greater White-Fronted Geese Along the East Asian Flyway

**DOI:** 10.3390/biology14081050

**Published:** 2025-08-14

**Authors:** Chunxiao Wang, Shaoxia Xia, Xiubo Yu, Houlang Duan, Guang Qi

**Affiliations:** 1 Key Laboratory of Ecosystem Network Observation and Modeling, Institute of Geographic Sciences and Natural Resources Research, Chinese Academy of Sciences, Beijing 100101, China; wangcx.19b@igsnrr.ac.cn (C.W.); duanhl@igsnrr.ac.cn (H.D.); 2 College of Resources and Environment, University of Chinese Academy of Sciences, Beijing 100049, China; 3 College of Chemistry and Environmental Engineering, Pingdingshan University, Pingdingshan 467000, China; qiguang594@163.com

**Keywords:** climate change, habitat suitability, species distribution modeling, greater white-fronted goose (*Anser albifrons frontalis*), east asian flyway

## Abstract

Migratory flyways sustain waterbird populations by linking critical habitats across their annual cycle. However, stage-specific impacts of climate change on these habitats remain poorly understood. We integrated species distribution models with annual migration data from 30 Greater White-fronted Geese (*Anser albifrons frontalis*) to assess changes in habitat suitability, distributional shifts, and suitability fluctuations across breeding, stopover, and wintering stages under mid-century (2040–2060) climate scenarios. Suitability fluctuations were quantified as the coefficient of variation ($C_{V}$) in habitat suitability between current and future projections. Projected habitat responses varied markedly across stages: breeding areas contracted by 29.9%, wintering areas expanded by 62.7%, and stopover sites showed minimal net change. Centroids of all habitats are projected to shift northward by mean distances of 125–492 km under future climate scenarios. Breeding habitats exhibited the greatest suitability fluctuations ($C_{V}=3$0–45; ~50% area affected under SSP585), followed by stopover and wintering grounds ($C_{V}$ ≈ 11), with 35.8% and 23.3% of their areas falling within high-fluctuation zones. These findings highlight the urgent need to prioritize breeding habitats, implement stage-specific conservation strategies, and enhance international cooperation to ensure the protection of waterbirds along the East Asian Flyway.

## Simple Summary

Migratory birds rely on networks of breeding, stopover, and wintering sites to complete their annual life cycle. However, climate change is rapidly shifting the distribution and suitability of these habitats, posing increasing risks to migratory species. In this study, we integrated satellite tracking data from 30 Greater White-fronted Geese (*Anser albifrons frontalis*) with year-round migration records, using species distribution models to assess how near-term climate change (2040–2060) may affect their habitats along the East Asian Flyway. Our results reveal a northward shift in suitable habitats, with significant losses in breeding areas (−29.9%), minor changes at stopover sites, and major expansions in wintering regions (+62.7%). Habitat suitability also showed strong fluctuations, particularly in breeding areas. These findings underscore the need for stage-specific conservation strategies and international cooperation to protect key habitats along the migratory flyway.

## 1. Introduction

Global surface temperatures have risen by an average of 1.1 °C since pre-industrial times [1], with recent years—including 2024—setting new records marked by more frequent heatwaves and accelerated glacial retreat [2,3,4]. Climate change is increasingly reshaping ecosystems [5], altering habitat structure and species distributions [6,7], disrupting migration patterns [8], and accelerating biodiversity loss [9,10,11]. Migratory waterbirds are among the taxa most affected, relying on distinct breeding, stopover, and wintering habitats along flyways to complete their annual cycle [12]. However, climate change is shifting the spatial distribution of these habitats, increasingly limiting access to critical resources across migratory stages [13]. While these changes are evident, their stage-specific consequences remain poorly understood. Given the ecological importance of migratory waterbirds and their reliance on connected flyway habitats [14,15], understanding these spatial dynamics is essential to guide effective conservation under climate change.

Most previous research has focused on isolated stages of the migratory cycle, such as breeding or wintering periods, to assess the impacts of climate change on habitat distribution and species behavior [16]. For example, many Arctic-breeding birds are shifting to higher latitudes or elevations in response to rising temperatures [17]. The advent of GPS tracking has enabled high-resolution spatiotemporal monitoring of bird movements across broad and remote regions [18], facilitating continuous monitoring throughout the entire annual migratory cycle. This technology has greatly improved our understanding of climate-driven changes in migration dynamics, habitat selection, and spatiotemporal habitat distribution [19,20].

Species distribution models (SDMs) have become essential tools for predicting potential species distributions under current and future climate scenarios, using environmental variables and occurrence data, thereby supporting assessments of habitat shifts [21]. Ensemble SDMs, which integrate multiple algorithms, improve prediction accuracy and robustness by reducing inter-model variability and enhancing the reliability of habitat forecasts [22]. While SDMs have been widely used to analyze habitat changes [23,24], recent advances have incorporated temporal dimensions into modeling, including phenology and demographic stages [25]. However, SDMs remain limited in capturing habitat dynamics across multiple stages of migratory birds’ annual cycles. To bridge this gap, we used the Greater White-fronted Goose (*Anser albifrons frontalis*, hereafter GWFG) as a focal subspecies to examine stage-specific habitat changes along the East Asian Flyway.

The East Asian Flyway, one of the world’s nine major migratory routes, supports millions of migratory birds, including the GWFG. According to the IUCN, the global population of GWFG is estimated at 5–6 million individuals, and the species is currently classified as “Least Concern” [26]. However, climate change is increasingly altering habitats along the GWFG’s migratory route. During extreme drought events, GWFG wintering sites have shifted from natural wetlands to artificial environments [27]. Climate-induced phenological mismatches, defined as temporal misalignments between the timing of biological events, such as food availability and bird migration, may also disrupt the connectivity of stopover habitats during migration [28]. Furthermore, rising temperatures are reducing the availability of suitable breeding habitats in the Arctic tundra, posing direct threats to breeding populations [29,30]. These stage-specific threats underscore the importance of assessing climate change impacts across the GWFG’s full migratory cycle.

We assessed the impacts of near-term climate change (2040–2060) on the GWFG habitats along the East Asian Flyway across breeding, stopover, and wintering stages. Using satellite tracking data and ensemble species distribution models, we simulated suitable habitats for each stage. Climate impacts were evaluated based on changes in habitat area, spatial shifts in habitat centroids, and temporal fluctuations in suitability. Our objective was to identify stage-specific differences in climate responses and inform targeted conservation efforts.

## 2. Materials and Methods

### 2.1. Occurrence Data

**Figure 1:**
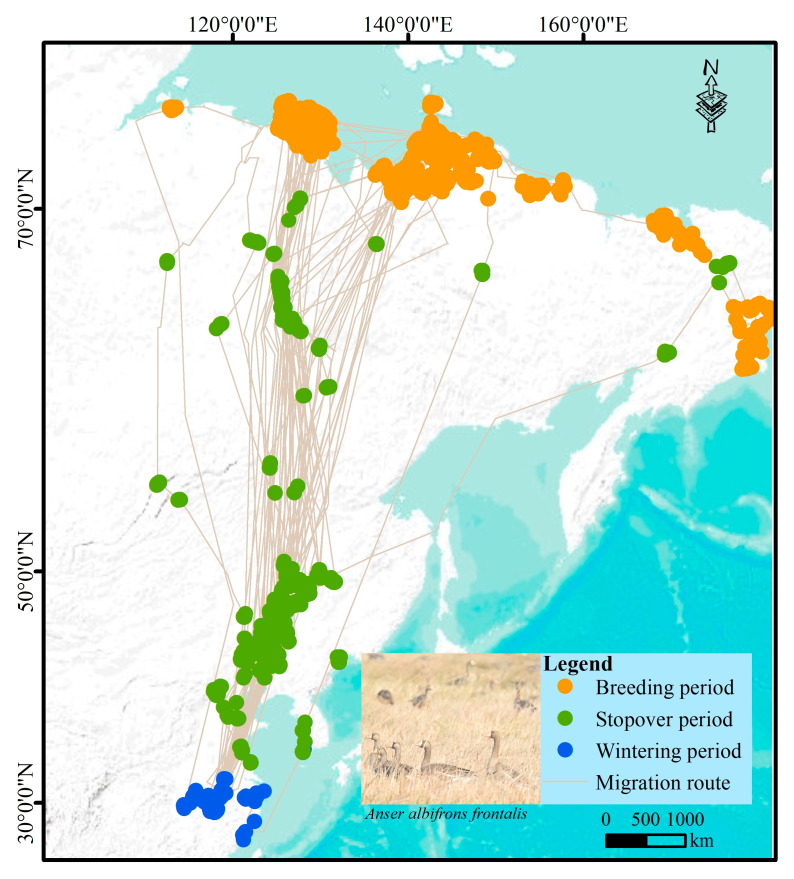
Satellite tracking data from 30 Greater White-fronted Geese (GWFG) used to model habitat use across breeding, stopover, and wintering periods. Migration routes are shown as lines con-necting consecutive locations for each individual. Basemap imagery provided by Esri (online basemap service). Photo copyright: Xiubo Yu.

Thirty-five GWFG were captured using mist nets and fitted with satellite transmitters (model HQBG3621; Hunan Global Messenger Technology Co., Ltd.; Changsha City, China) in a backpack configuration secured with Teflon ribbon. All captures were conducted non-destructively during the winters of 2018 and 2019 at the Nanji Wetland National Nature Reserve of Poyang Lake (28°51′ N, 116°10′ E), a key wintering site for the subspecies *Anser albifrons frontalis*; thus, the tracked individuals likely represent the primary GWFG population wintering in the Yangtze River Basin [31]. The devices weighed 26 ± 3 g (<3% of body mass, 1400–2300 g), minimizing potential impacts on behavior or energetics [32]. The trackers recorded hourly data on timestamp, location (latitude and longitude), and related metadata. Although 35 individuals were initially tagged, only 30 with complete high-resolution annual migration data were retained for analysis, with the remainder excluded due to transmitter failure, mortality, or incomplete coverage of the full migration cycle (Figure 1; see Supplementary Table S1).

We preprocessed the tracking data to reduce spatial and temporal autocorrelation, as required by SDMs [33]. First, we excluded locations with a positional error exceeding 100 m. To identify and remove points associated with in-flight behavior, we then applied the Expectation-Maximization binary Clustering (EMbC) algorithm [33], which classifies movement trajectories into behavioral modes based on speed and turning angle. The remaining points were classified into three migratory stages based on timing: breeding (days 180–270), wintering (days 320–120 of the following year), and stopover (locations used for more than two consecutive days outside the breeding and wintering periods) [34,35]. To further minimize spatial autocorrelation, we randomly selected one point per 1 km^2^ grid cell from the pooled dataset across all individuals [36]. This filtering procedure yielded 6213 occurrence points for the breeding period, 4188 for stopovers, and 5738 for the wintering period.

### 2.2. Environmental Data

We considered four categories of annual environmental factors for GWFG distribution: climate, land use, topography, and human impact. While these variables do not reflect monthly or seasonal changes, they are appropriate for assessing broad-scale habitat responses to future environmental trends. Climate data included 19 bioclimatic variables from WorldClim v2.1 at 30-arc-second (~1 km) resolution, representing averages for 1970–2000 (www.worldclim.org, accessed on 8 January 2025). Land use data were derived from GlobeLand30, providing 30 m resolution coverage across the entire flyway (www.webmap.cn, accessed on 8 January 2025). Topographic variables (elevation and slope) at 1 km resolution were obtained from EarthEnv (www.earthenv.org, accessed on 10 January 2025). Human impact was represented by the Global Human Footprint Dataset at 1 km resolution [37].

The 2040–2060 period from WorldClim’s CMIP6 projections was selected to represent future climate conditions, as it aligns with standardized mid-century benchmarks and supports assessment of near-term conservation challenges with reduced long-term uncertainty. We selected the BCC-CSM2-MR model for its robustness and suitability in capturing East Asian climate dynamics [38]. Projections were based on three Shared Socioeconomic Pathways (SSPs): SSP126 (low), SSP245 (moderate), and SSP585 (high emissions). Future land use for 2050, derived from these scenarios, was simulated using GeoSOS-FLUS at 1 km resolution (www.geosimulation.cn, accessed on 10 January 2025). These projections reflect spatial land use transitions under different SSPs and were directly incorporated into the SDMs to represent anthropogenic landscape change, thereby indirectly capturing land use dynamics.

All variables were resampled to a common 1 km spatial resolution and coordinate system. Multicollinearity among environmental variables can inflate model uncertainty and reduce predictive performance [39]. We addressed this by using the “vifstep” function from the usdm package in R (version 4.4.2), retaining variables with low multicollinearity (*VIF* < 6; |*r*| < 0.8) (see Supplementary Table S2 and Figure S1) [40]. In total, 10, 9, and 12 predictors were retained for the breeding, stopover, and wintering periods, respectively (Table 1).

### 2.3. SDM Analysis

To model the habitat distribution of GWFG during breeding, stopover, and wintering periods, we employed a regression-based method (generalized linear model, GLM) alongside three machine learning algorithms—boosted regression trees (BRT), random forests (RF), and support vector machines (SVM). These models integrate the strengths of extrapolative and interpolative methods, offering complementary insights across ecological relationships [41]. Model performance was evaluated using eight bootstrap iterations, each involving a random split of 70% training and 30% testing data. For each migration stage, 5000 background points were randomly sampled to represent the environmental background. All models were implemented independently for each migration stage using the “sdm” package in R 4.1.2 [40].

Model performance was assessed using the area under the receiver operating characteristic curve (AUC) and the true skill statistic (TSS), with AUC values approaching 1 and TSS values near 1 indicating high predictive accuracy. To reduce uncertainty and improve robustness, we constructed ensemble models by weighting individual algorithms according to their TSS scores.

To quantify the relative importance of environmental variables across migration stages, we applied a permutation-based importance analysis using both Pearson correlation and AUC-based metrics. The average importance of each variable was then computed across all model types. Finally, the models were projected onto mid-century climate scenarios to predict future habitat suitability under changing climatic conditions.

### 2.4. Analysis of Habitat Change Under Climate Change

The potential habitat maps for GWFG were generated using the optimal threshold determined by the sensitivity–specificity sum maximization approach [42]. These thresholds, distinguishing suitable from unsuitable habitats, were 0.42 for breeding grounds, 0.60 for stopover sites, and 0.31 for wintering grounds. Subsequently, we quantified the projected impacts of climate change on GWFG habitats across the three migratory stages using three indicators: potential habitat area, shifts in habitat centroids, and fluctuations in habitat suitability.

We calculated the current and projected suitable habitat areas and compared the areas of habitat loss and gain under three climate scenarios for the period 2040–2060. Next, we used ArcGIS 10.8 to calculate the habitat centroids and illustrate the spatial shifts (direction and distance) of these centroids for GWFG under different climatic scenarios [43]. Finally, we assessed habitat suitability fluctuations within current habitat boundaries under future climate conditions using the coefficient of variation ($C_{V}$), defined as:

(1) $$C_{V}\;=\;\frac{\sigma }{\mu }$$

where σ is the standard deviation and μ is the arithmetic mean of habitat suitability [44]. Higher $C_{V}$ values indicate greater habitat fluctuation. Based on natural breaks in habitat suitability variability, we classified all habitats into two categories: low-fluctuation and high-fluctuation zones.

### 2.5. Statistical Analysis

To assess the significance of habitat changes across migratory stages, we used one-way ANOVA with Tukey’s HSD tests. The independent variable was habitat during the breeding, stopover, and wintering periods, while the dependent variables were habitat area, centroid shift angle and distance, and suitability fluctuation values. Each migratory stage was represented by mean values across three climate scenarios. All statistical analyses were performed using Origin 2024.

## 3. Results

### 3.1. Environmental Drivers of Breeding, Stopover, and Wintering Habitat Distribution

The GLM, BRT, RF, and SVM models for GWFG across different migratory stages achieved high predictive performance, with mean AUC and TSS values exceeding 0.9 and standard deviations below 0.32 (see Supplementary Figures S2 and S3).

**Figure 2:**
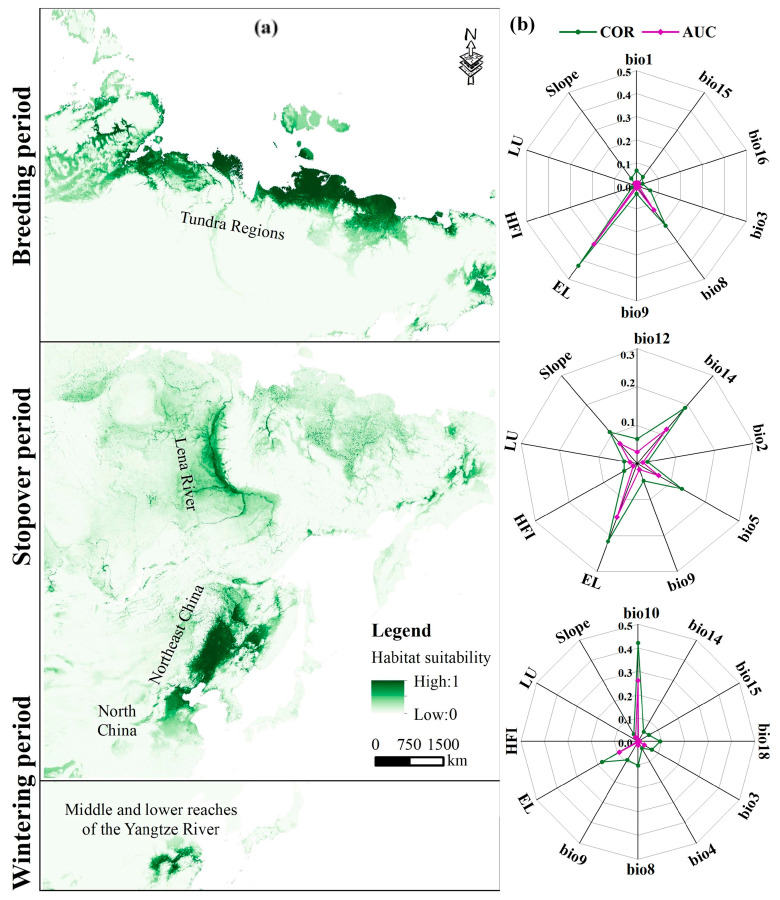
(**a**) Predicted habitat suitability for GWFG during the breeding, stopover, and wintering stages under current climate conditions; (**b**) relative contributions of environmental variables to habitat distribution, assessed using Pearson correlation and AUC-based importance metrics.

The current breeding range of the GWFG covers approximately 1,928,000 km^2^, primarily spanning the tundra regions of northern Russia, from Severnaya Zemlya to the Anadyr River (Figure 2a). Stopover habitats extend across approximately 1,212,000 km^2^, encompassing wetlands along the Bohai Sea, river and lake wetlands in Northeast China (including Moonpao, Wudalianchi, Nenjiang, and the Tumen River), coastal wetlands in western Korea, Lake Baikal, and the Lena River Basin. Wintering habitats cover approximately 260,000 km^2^, mainly in the wetlands of the middle and lower Yangtze River, including Poyang Lake and Dongting Lake.

Breeding habitat distribution is predominantly driven by elevation (EL) and mean temperature of the wettest quarter (bio8) (Figure 2b). Stopover habitats are influenced primarily by EL, precipitation of the driest month (bio14), maximum temperature of the warmest month (bio5), and slope. Wintering habitats are largely affected by the mean temperature of the warmest quarter (bio10) and EL.

### 3.2. Changes in Area and Distribution of Suitable Habitat Under Future Climate Change

**Figure 3:**
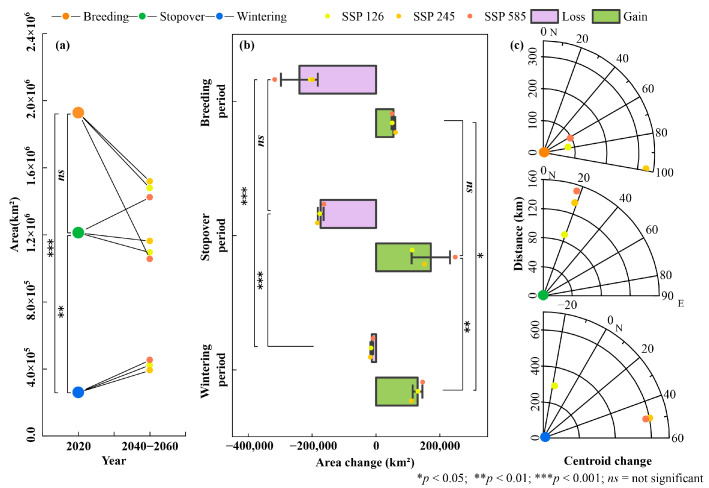
(**a**) Potential suitable habitat areas for the breeding, stopover, and wintering periods under current and future climate scenarios; (**b**) habitat area gain and loss under future scenarios, including significance analysis across different periods; (**c**) habitat centroid shift angles and distances in future scenarios, where the center of the sector represents the current habitat centroid, the radius indicates the shift distance, and the arc length represents the shift angle.

Based on habitat distributions under future scenarios (see Supplementary Figure S4), significant area changes were observed across the three migratory stages (F = 38.63, *p* < 0.001). Breeding habitats decreased significantly, while wintering habitats increased notably. Changes in stopover habitats varied across climate scenarios (Figure 3a). On average, habitat areas changed by −29.93% for breeding, +1.24% for stopover, and +62.67% for the wintering stages. Under the high-emission scenario SSP585, breeding habitats experienced the largest decrease, losing 316,843 km^2^, while wintering habitats experienced the largest increase, gaining 146,964 km^2^. In the same scenario, stopover habitats exhibited a net increase, with gains of 249,145 km^2^ surpassing losses of 162,274 km^2^ (Figure 3b). Habitat gains and losses showed statistically significant differences (gain: F = 69.72, *p* < 0.001; loss: F = 13.47, *p* < 0.05).

Under the three future climate scenarios, habitats of all stages shifted northward, with no significant differences in the shift angles across the different stages (F = 5.39, *p* = 0.0525). However, the shift distances varied significantly among the stages (F = 7.36, *p* < 0.05) (Figure 3c). Specifically, breeding, stopover, and wintering habitat centroids shifted northward by 79.9–327.1 km (mean ≈ 168 km), 89.0–151.2 km (mean ≈ 125 km), and 295.5–602.4 km (mean ≈ 492 km), respectively. These values indicate that centroid shifts of wintering habitats were substantially larger than those of breeding or stopover habitats.

**Figure 4:**
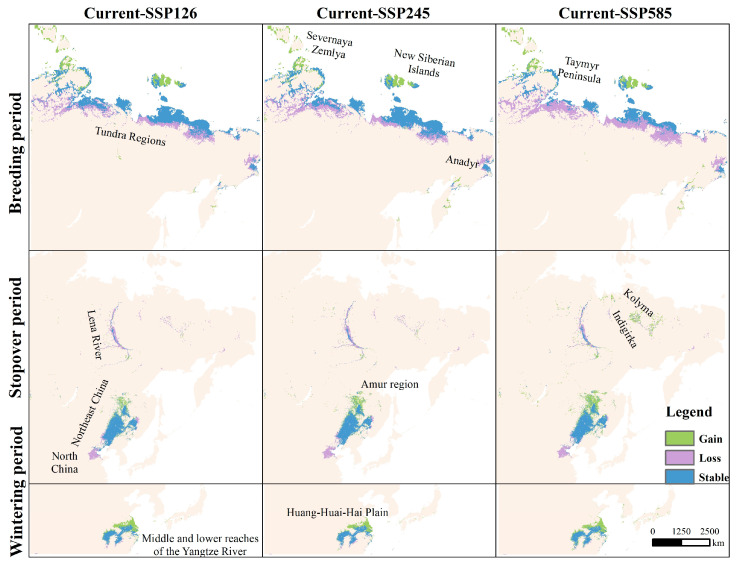
Distribution of gain, loss, and stable areas of potential habitats for the breeding, stopover, and wintering periods under the three future climate scenarios compared to current conditions.

Suitable breeding habitats in the tundra are projected to decline, while coastal wetlands in the New Siberian Islands and Severnaya Zemlya are expected to expand (Figure 4). Stopover habitats are likely to shrink in the North China Plain and Lena River wetlands, while new habitats may emerge in the Amur region and the lowlands of the Indigirka and Kolyma Rivers. Wintering grounds are predicted to shift northward, expanding into regions such as the Huang-Huai-Hai Plain wetlands. Overall, current GWFG habitats are expected to contract in southern areas and expand in northern ones as a response to future climate change.

### 3.3. Habitat Suitability Fluctuation Under Future Climate Change

**Figure 5:**
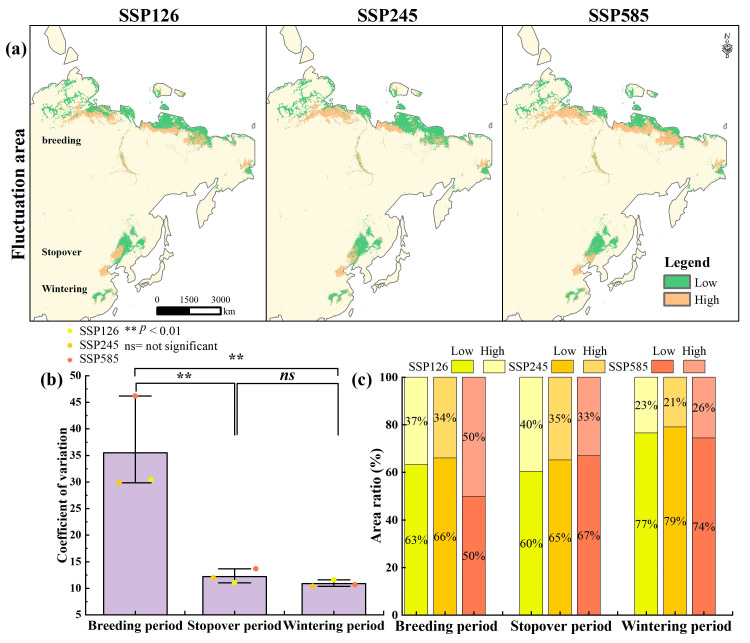
(**a**) The distribution of high and low habitat suitability fluctuation zones under future climate scenarios, (**b**) coefficient of variation values across different stages, and (**c**) the percentage of current habitat area occupied under high and low fluctuation zones.

High habitat suitability fluctuations were predominantly associated with areas of projected habitat loss, particularly along the southern margins of current ranges (Figure 5a). During the breeding period, $C_{V}$ values ranged from 30 to 45, with approximately 50% of the area classified as highly variable under the high-emission scenario SSP585 (Figure 5b,c). In contrast, mean $C_{V}$ values during the stopover and wintering periods were approximately 11, with 35.8% and 23.3% of the respective areas exhibiting high variability. Overall, habitat suitability fluctuations were most pronounced during the breeding stage, followed by the stopover and wintering stages (F = 19.64, *p* < 0.01).

## 4. Discussion

### 4.1. Habitat Distribution and Key Environmental Factors

We used tracking data and an ensemble of species distribution models to assess potential suitable habitats along the East Asian Flyway. Our findings are consistent with previous research, indicating that the breeding grounds span from Severnaya Zemlya to the Anadyr River, reflecting a Siberian distribution within the Palaearctic [45]. The stopover sites form a distinctive sickle-shaped corridor along the Lena River and extend across the Northeastern Plain [46], while the wintering grounds are located in the middle and lower reaches of the Yangtze River [34].

GWFG habitat distribution during different migratory stages is influenced by distinct environmental factors. First, our results demonstrate that elevation is a significant factor at all stages (Figure 2b), with GWFG showing a clear preference for potential habitats at lower elevations and gentler slopes [47], as lower areas near the water provide them with optimal food resources [48]. Even when confronted with ecological barriers during migration, the birds strategically navigate detours to maintain lower elevation pathways [49]. Second, temperature is a key factor in habitat distribution [50]. During the wintering and stopover periods, geese prefer warmer habitats [51], consistent with our findings that wintering habitats are influenced by the mean temperature of the warmest quarter (bio10) and stopover habitats by the maximum temperature of the warmest month (bio5). This may be due to the temperature during the warmest month affecting vegetation germination and phenology. Additionally, breeding habitats are shaped by the mean temperature of the wettest quarter (bio8), consistent with previous studies indicating that temperature and precipitation are key determinants of geese breeding habitats [29]. These factors may affect habitat suitability by altering vegetation growth and phenological matching [52]. Finally, the precipitation of the driest month (bio14) also influences stopover site distribution by maintaining wetland hydrology, which provides essential water and food resources for migrating geese [53].

### 4.2. Effects of Climate Change on Habitat Distribution and Stability

Our findings suggest that the habitat distribution of the GWFG is projected to shift northward along its migratory flyway in response to climate change, consistent with similar poleward movements observed among migratory species worldwide [54,55]. However, the effects of climate change on GWFG habitats differ substantially across the breeding, stopover, and wintering stages of their annual cycle.

Breeding habitats are projected to undergo the most severe climate-driven changes among all migratory stages. Specifically, suitable areas are expected to decline by 29.9%, accompanied by a northward shift of up to 327 km (mean = 168 km). This shift is likely driven primarily by declining habitat suitability in the southern tundra, where shrub encroachment is reducing open-ground breeding areas [30,56]. However, further northward expansion is constrained by the Arctic coastline, likely confining breeding to a narrow latitudinal band and shortening the breeding window [57]. In addition to spatial contraction, breeding habitats also showed the greatest instability ($C_{V}$ = 30–45), with approximately 50% of the area classified as highly variable under SSP585. Driven by climate warming, the contraction and instability of breeding habitats, especially at southern edges, may disrupt food availability and nest-site quality, ultimately compromising reproductive success.

Stopover habitats may either increase or decrease depending on the specific climate scenarios. This variability in stopover habitats is likely due to the region’s mid-latitude location in East Asia, where temperature and precipitation fluctuate significantly [58]. Among these areas, the Northeast Plain is a key staging region for GWFG, supporting critical refueling during migration and sustaining flyway connectivity [46,49]. However, our results indicate that stopover habitats are shifting northward and exhibit elevated suitability fluctuations (mean $C_{V}$ ≈ 11; 35.8% of the area highly variable). These fluctuations may disrupt resource availability and flyway connectivity, potentially compromising refueling efficiency and migration success.

Wintering habitats are projected to also shift northward, with newly suitable areas emerging in the Huang-Huai-Hai region beyond the current range. This pattern aligns with recent observations of population redistribution, such as declines at Dongting and Poyang Lakes and increases at more northerly sites like Shengjin Lake [46,49]. However, despite the projected expansion in wintering habitats, SDMs do not incorporate habitat quality or resource availability, which are central to actual site use by geese. In reality, newly suitable regions face rapid land use changes, such as agricultural expansion and wetland reclamation, which have led to significant habitat degradation [59,60]. Moreover, increasing frequency of extreme droughts in the Yangtze River Basin has disrupted food availability in core wintering sites. These changes pose critical threats to GWFG populations, which rely heavily on natural wetlands for overwintering [61].

We also found that projected habitat responses differed between SSP126, SSP245, and SSP585, reflecting scenario-specific ecological impacts. For example, breeding habitats showed the greatest reduction under the high-emission scenario (SSP585), while losses were notably lower under both the low-emission (SSP126) and intermediate (SSP245) scenarios. In addition to variations across emission scenarios, temporal differences in projected impacts may also influence conservation outcomes. Specifically, we acknowledge that habitat responses may differ under alternative future windows, such as the 2080s. These differences may arise from nonlinear ecological feedback and the accumulation of anthropogenic pressures over longer timescales [62].

### 4.3. Conservation Strategies for Greater White-Fronted Geese

Climate change should be integrated into conservation planning [63]. Priority should be given to protecting newly emerging northern habitats, such as wetlands in the Amur River basin and northeastern Siberia. In contrast, lower-emission trajectories offer a critical window for proactive adaptation and restoration within existing core habitats like the Yangtze River floodplain. To enhance effectiveness, conservation strategies should incorporate multi-scale temporal frameworks, including monthly or seasonal environmental variability as well as longer-term future timeframes. This approach can help mitigate negative impacts and sustain suitable habitats for migratory species like the GWFG under changing climate conditions.

As climate change does not affect all stages equally, a one-size-fits-all conservation approach is unlikely to be effective. Stage-specific strategies are needed to ensure that habitat protection is tailored to the unique conditions and requirements of each stage of the migratory cycle.

Breeding habitats are undergoing significant contraction and instability under climate change, and most of them are located in remote Arctic regions that are difficult to access for fieldwork. This makes it essential to develop automated monitoring tools, such as satellite trackers, bioacoustics, and video cameras [64,65,66]. These systems enable long-term tracking of population changes, nesting activity, and local environmental conditions, providing critical data to assess climate impacts on reproduction. Stopover sites in Northeast China show significant habitat fluctuations, with some wetlands—such as the Sanjiang Plain wetlands, West Liao River wetlands, and the Hongshan Reservoir—at risk of loss. Water level and microtopography modifications should be applied to restore these degraded wetlands, enhance waterbody aggregation, and maintain habitat connectivity along the flyway [47,67]. Although wintering habitats may expand under future climate scenarios, this does not guarantee improved habitat quality and resource availability. Without effective safeguards, these gains may be undermined by land use pressures and climate extremes. Conservation should therefore focus on protecting remaining natural wetlands and promoting low-disturbance alternatives, such as undisturbed rice paddies and restored floodplain wetlands, to buffer against climate-driven resource shortages [68].

While each stage—breeding, stopover, and wintering—is vital to the annual migration cycle, stage-specific actions must be integrated into a broader, coordinated conservation framework across the entire migratory corridor [69]. For instance, the East Asian–Australasian Flyway Partnership (https://eaaflyway.net/, accessed on 11 March 2025) connects countries and regions along the East Asian Flyway and plays a critical role in conserving habitats for the GWFG. As a key wintering and stopover country for this population, China must establish a national habitat protection network to help reverse population declines [67]. In 2024, the Chinese government launched the “China Action Plan for Migratory Bird Flyway Protection and Restoration (2024–2030),” a major policy milestone with strong potential to enhance coordinated conservation of the GWFG and other migratory species.

### 4.4. Limitations and Future Work

Although satellite tracking provides a novel method for studying the potential habitats of the GWFG across the East Asian Flyway, our study has several limitations that deserve consideration. All tracking devices were deployed at a single wintering site, Poyang Lake Wetland [70]. While this approach is logistically efficient and representative of the primary GWFG population wintering in China, it may lead to sampling bias due to the absence of data from other wintering populations (e.g., Republic of Korea) [71].

This spatial bias could potentially affect the accuracy of stage-specific habitat inferences, particularly during breeding and stopover periods. Our predicted breeding range, spanning from Severnaya Zemlya to the Anadyr River, shows broad agreement with established core breeding areas, although it may underrepresent marginal or less commonly used sites [45]. Northeast China is also an important stopover region for GWFG populations that winter in the Korean Peninsula. However, other stopover sites along their migratory flyways may not have been identified in our study [49]. Therefore, our results are most robust for the GWFG population wintering in the Yangtze River Basin and should be extrapolated to other wintering populations within the East Asian Flyway with caution.

To reduce spatial sampling bias and better capture population-level variation in responses to climate change, future research should extend satellite tracking to GWFG individuals from additional wintering populations (e.g., the Korean Peninsula, Japan). Integrating these efforts with ground-based monitoring of populations, habitat use, and citizen science observations will provide more comprehensive and continuous data along the East Asian Flyway, enabling a finer-scale understanding of migratory strategies and their sensitivity to environmental change.

## 5. Conclusions

We assessed the potential impacts of climate change on GWFG habitats across the breeding, stopover, and wintering stages along the East Asian Flyway. Our findings show that habitat suitability during each migratory stage is influenced by distinct environmental drivers. While all stages are projected to shift northward under future climate conditions, substantial differences were found in habitat area changes, shift distances, and suitability fluctuations across stages. In general, breeding habitats are expected to be most negatively affected, followed by stopover sites, while wintering habitats are projected to remain comparatively stable. To ensure the long-term survival of this migratory species, conservation efforts should include integrating climate considerations, enhancing international cooperation, and implementing stage-specific strategies such as breeding habitat monitoring, stopover site restoration, and reduction of human disturbance in wintering areas.

## Figures and Tables

**Table 1:** Environmental variables used in species distribution models across the three migratory stages.

Migration Stage	Variable Code	Variable Name	Unit
Breeding period	bio1	Annual mean temperature	°C
	bio15	Precipitation seasonality (coefficient of variation)	%
	bio16	Precipitation of wettest quarter	mm
	bio3	Isothermality (bio2/bio7) (×100)	/
	bio8	Mean temperature of wettest quarter	°C
	bio9	Mean temperature of driest quarter	°C
	EL	Elevation	m
	HFI	Human footprint index	/
	LU	Land use	/
	Slope	Slope	%
Stopover period	bio12	Annual precipitation	mm
	bio14	Precipitation of driest month	mm
	bio2	Mean diurnal range (mean of monthly (max temp − min temp))	°C
	bio5	Max temperature of warmest month	°C
	bio9	Mean temperature of driest quarter	°C
	EL	Elevation	m
	HFI	Human footprint index	/
	LU	Land use	/
	Slope	Slope	%
Wintering period	bio10	Mean temperature of warmest quarter	°C
	bio14	Precipitation of driest month	mm
	bio15	Precipitation seasonality (coefficient of variation)	%
	bio18	Precipitation of warmest quarter	mm
	bio3	Isothermality (bio2/bio7) (×100)	/
	bio4	Temperature seasonality (standard deviation × 100)	°C
	bio8	Mean temperature of wettest quarter	°C
	bio9	Mean temperature of driest quarter	°C
	EL	Elevation	m
	HFI	Human footprint index	/
	LU	Land use	/
	Slope	Slope	%

## Data Availability

Data will be available on request.

## Supplementary Materials

The following supporting information can be downloaded at: https://www.mdpi.com/article/10.3390/biology14081050/s1, Table S1. Summary of migration data from 30 Greater White-fronted Geese (*Anser albifrons frontalis*) used to obtain the species occurrence records; Table S2. Environmental variables filtered based on correlation coefficient and variance inflation factors (*VIF*); Figure S1. Statistical description and correlation coefficients between environmental variables. * Means significant at 95% confidence level (*p* < 0.05). ** Means significant at 99% confidence level (*p* < 0.01); Figure S2. Mean AUC and TSS for GWFG (*Anser albifrons frontalis*) predicted by GLM, BRT, RF, and SVM models. Abbreviations: GLM—Generalized Linear Model, BRT—Boosted Regression Trees, RF—Random Forest, SVM—Support Vector Machine, AUC—Area Under the Curve (ROC), TSS—True Skill Statistic, GWFG—Greater White-fronted Geese; Figure S3. Occurrence points and habitat distribution of GWFG across three migratory stages under current climatic conditions using different models; Figure S4. Predicted habitat distribution of GWFG under future climate scenarios.

## Abbreviations

GWFG	Greater White-fronted Goose
SDM	Species distribution model
GLM	Generalized Linear Model
BRT	Boosted Regression Trees
RF	Random Forest
SVM	Support Vector Machine
AUC	Area Under the Curve
TSS	True Skill Statistic
