# Supplementary material for: Stage-Specific Impacts of Climate Change on Greater White-Fronted Geese Along the East Asian Flyway
Source: Biology (Basel). 2025 Aug 14;14(8):1050. doi: 10.3390/biology14081050 (PMC12383389; doi:10.3390/biology14081050)
Supplement: Supplementary file 1 [file biology-14-01050-s001.zip › biology-3783198-supplementary.docx]

**Stage-specific impacts of climate change on Greater White-fronted Geese along the East Asian Flyway**

**Table S1.** Summary of migration data from 30 Greater White-fronted Goose (*Anser albifrons frontalis*) used to obtain the species occurrence records.

| **Serial number** | **GPS tracker ID** | **Start time** | **End time** | **Number of sites** |
| --- | --- | --- | --- | --- |
| 1 | BFUL219 | 2018/03/19 00:01 | 2019/03/19 20:07 | 6,274 |
| 2 | BFUL222 | 2018/03/19 00:01 | 2019/03/19 07:01 | 5,929 |
| 3 | BFUL224 | 2018/03/19 00:00 | 2019/03/19 22:00 | 6,677 |
| 4 | BFUL271 | 2018/10/22 15:22 | 2019/10/22 23:00 | 6,370 |
| 5 | BFUL273 | 2018/10/22 15:17 | 2019/10/22 23:00 | 6,461 |
| 6 | BFUL274 | 2018/10/22 15:15 | 2019/10/22 18:02 | 6,187 |
| 7 | BFUL275 | 2018/10/22 15:30 | 2019/10/22 22:00 | 6,044 |
| 8 | BFUL280 | 2018/10/22 15:25 | 2019/10/22 21:01 | 5,821 |
| 9 | BFUL289 | 2018/10/22 15:09 | 2019/10/17 00:01 | 3,364 |
| 10 | BFUL290 | 2018/10/22 14:59 | 2019/10/22 22:00 | 3,859 |
| 11 | BFUL293 | 2018/10/22 14:55 | 2019/10/22 22:00 | 6,113 |
| 12 | GSNR059 | 2018/01/01 04:00 | 2019/01/01 12:00 | 7,793 |
| 13 | GSNR097 | 2018/10/22 14:19 | 2019/10/22 21:06 | 5,014 |
| 14 | GSNR098 | 2018/10/22 15:20 | 2019/10/22 22:00 | 7,472 |
| 15 | GSNR104 | 2018/10/22 16:01 | 2019/10/22 22:00 | 6,373 |
| 16 | GSNR108 | 2018/10/22 15:13 | 2019/10/22 22:00 | 6,713 |
| 17 | GSNR111 | 2018/10/22 15:18 | 2019/10/22 22:00 | 6,553 |
| 18 | GSNR116 | 2018/10/22 14:39 | 2019/10/21 20:49 | 6,752 |
| 19 | GSNR118 | 2018/10/22 15:20 | 2019/10/22 22:00 | 6,750 |
| 20 | GSNR119 | 2018/10/22 14:54 | 2019/10/22 22:00 | 8,038 |
| 21 | GSNR121 | 2019/03/05 10:40 | 2020/03/05 22:00 | 6,578 |
| 22 | GSNR123 | 2019/03/05 10:33 | 2020/03/05 23:00 | 8,339 |
| 23 | GSNR131 | 2019/03/05 11:00 | 2020/03/05 22:00 | 7,141 |
| 24 | GSNR136 | 2019/03/05 10:35 | 2020/02/18 06:01 | 3,968 |
| 25 | GSNR138 | 2019/03/05 10:39 | 2020/03/05 22:00 | 8,248 |
| 26 | GSNR139 | 2019/03/05 10:38 | 2020/03/05 22:00 | 8,385 |
| 27 | GSNR144 | 2019/03/05 10:31 | 2020/03/05 22:00 | 6,426 |
| 28 | GSNR145 | 2019/03/05 10:44 | 2020/03/05 22:00 | 8,385 |
| 29 | GSNR148 | 2019/03/05 10:34 | 2020/03/05 22:00 | 8,296 |
| 30 | GSNR150 | 2019/03/05 10:36 | 2020/05/03 22:00 | 8,003 |

**Table S2.** Environmental variables filtered based on correlation coefficient and variance inflation factors (*VIF*)

| migration period | environmental variables and VIF | | | | | | | | | | | | |
| --- | --- | --- | --- | --- | --- | --- | --- | --- | --- | --- | --- | --- | --- |
| breeding period | bio1  2.09 | bio15  2.21 | bio16  3.22 | bio3  2.24 | bio8  2.51 | bio9  2.45 | EL  1.83 | HFI  1.09 | LU  1.06 | Slope  1.75 |  |  |  |
| stopover period | bio12  1.87 | bio14  2.82 | bio2  2.33 | bio5  5.16 | bio9  2.85 | EL  1.76 | HFI  1.85 | LU  1.10 | Slope  1.23 |  |  |  |  |
| wintering period | bio10  4.51 | bio14  3.91 | bio15  3.35 | bio18  2.73 | bio3  1.99 | bio4  3.10 | bio8  1.86 | bio9  5.53 | EL  4.56 | HFI  1.46 | LU  1.25 | Slope  5.15 |  |


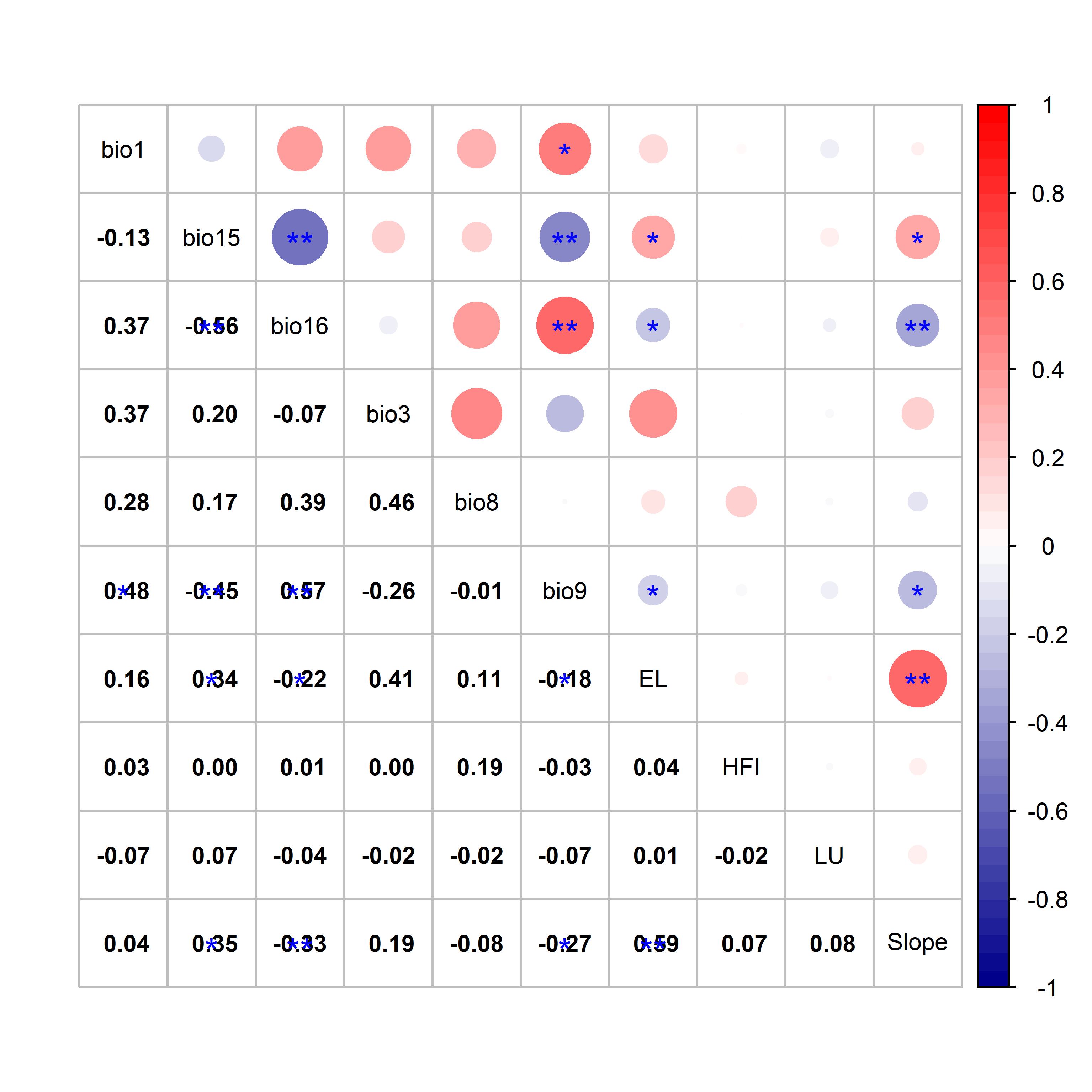

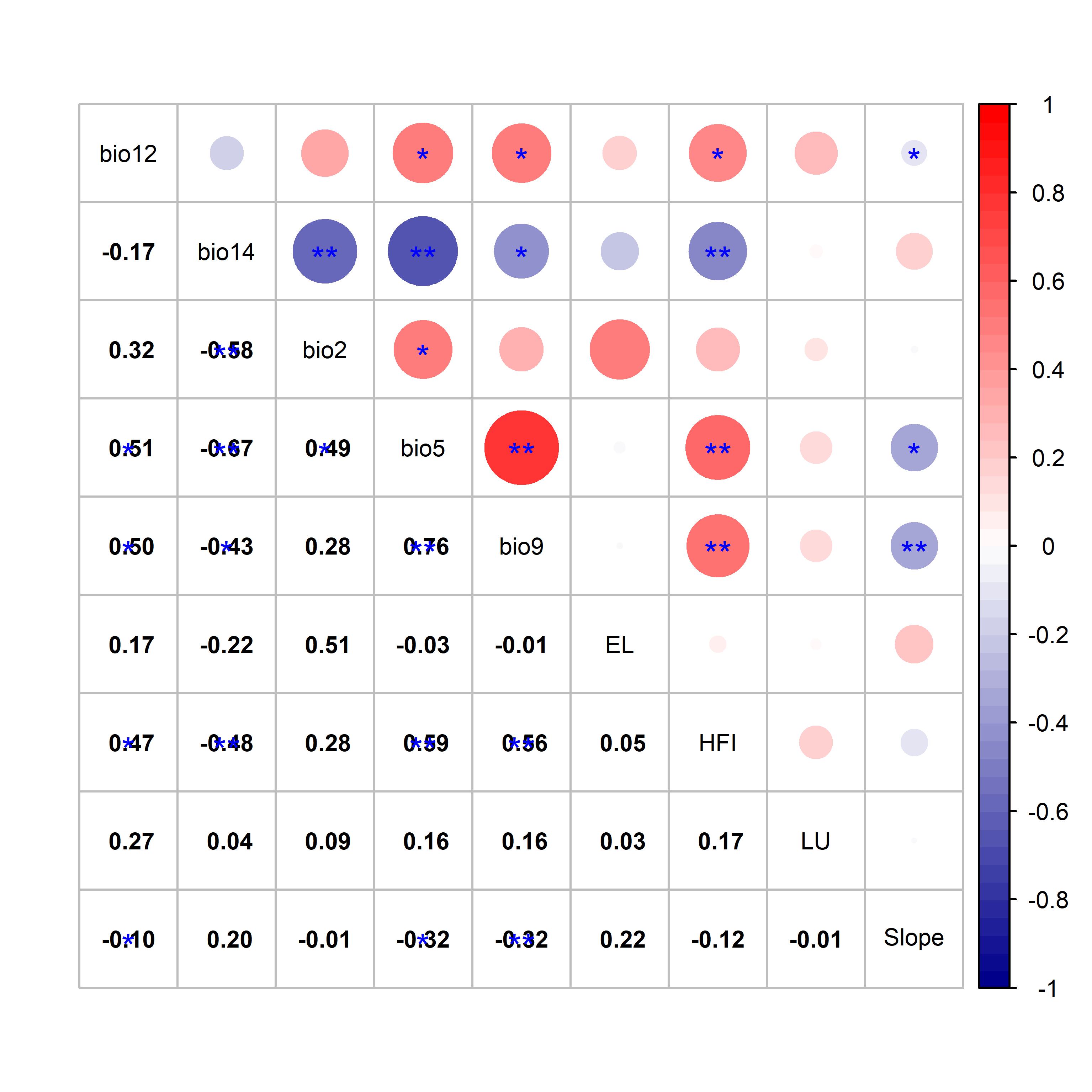

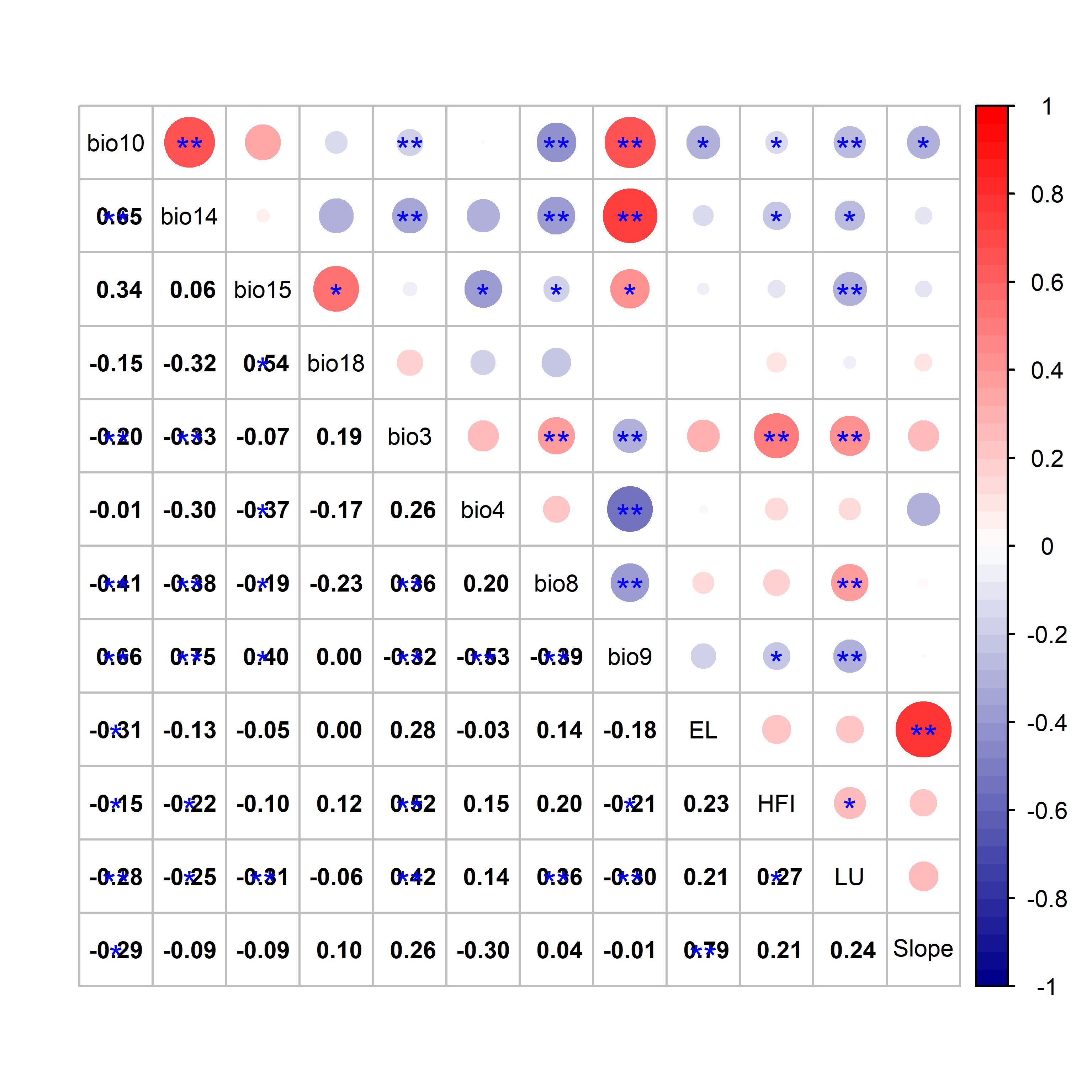


**Figure S1.** Statistical description and correlation coefficients between environmental variables. *Means significant at 95% confidence level (*p* < 0.05). **Means significant at 99% confidence level (*p* < 0.01).


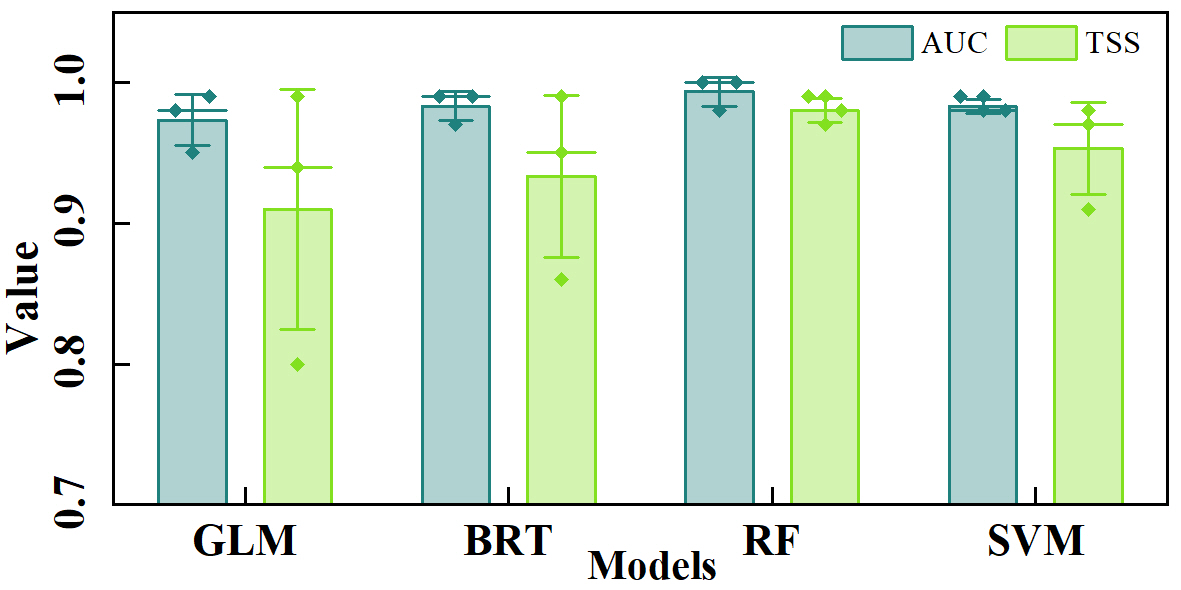


**Figure S2.** Mean AUC and TSS for GWFG (*Anser albifrons frontalis*) predicted by GLM, BRT, RF, and SVM models. Abbreviations: GLM - Generalized Linear Model, BRT - Boosted Regression Trees, RF - Random Forest, SVM - Support Vector Machine, AUC - Area Under the Curve (ROC), TSS - True Skill Statistic, GWFG - Greater White-fronted Geese


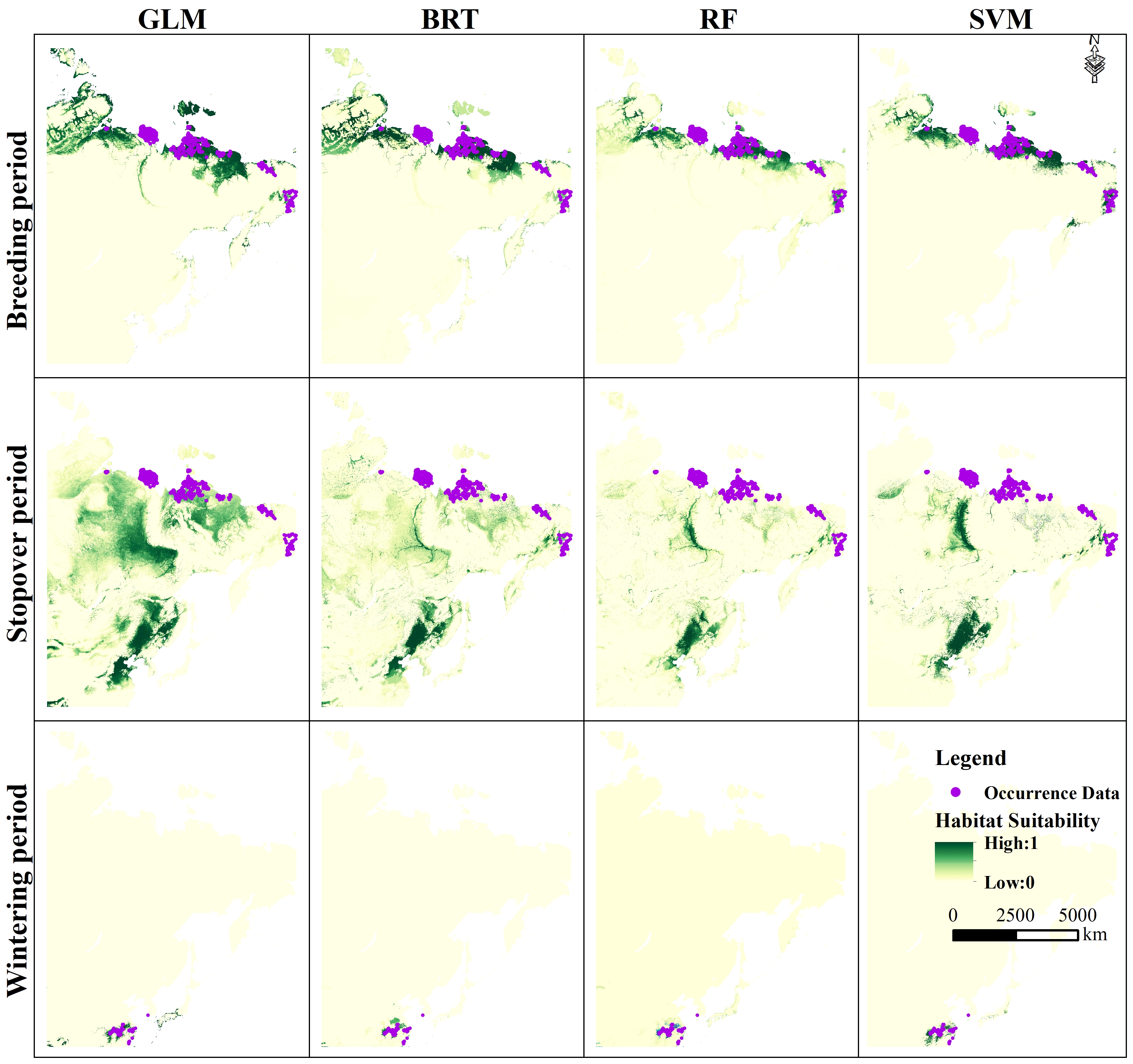


**Figure S3.** Occurrence points and habitat distribution of GWFG across three migratory stages under current climatic conditions using different models.


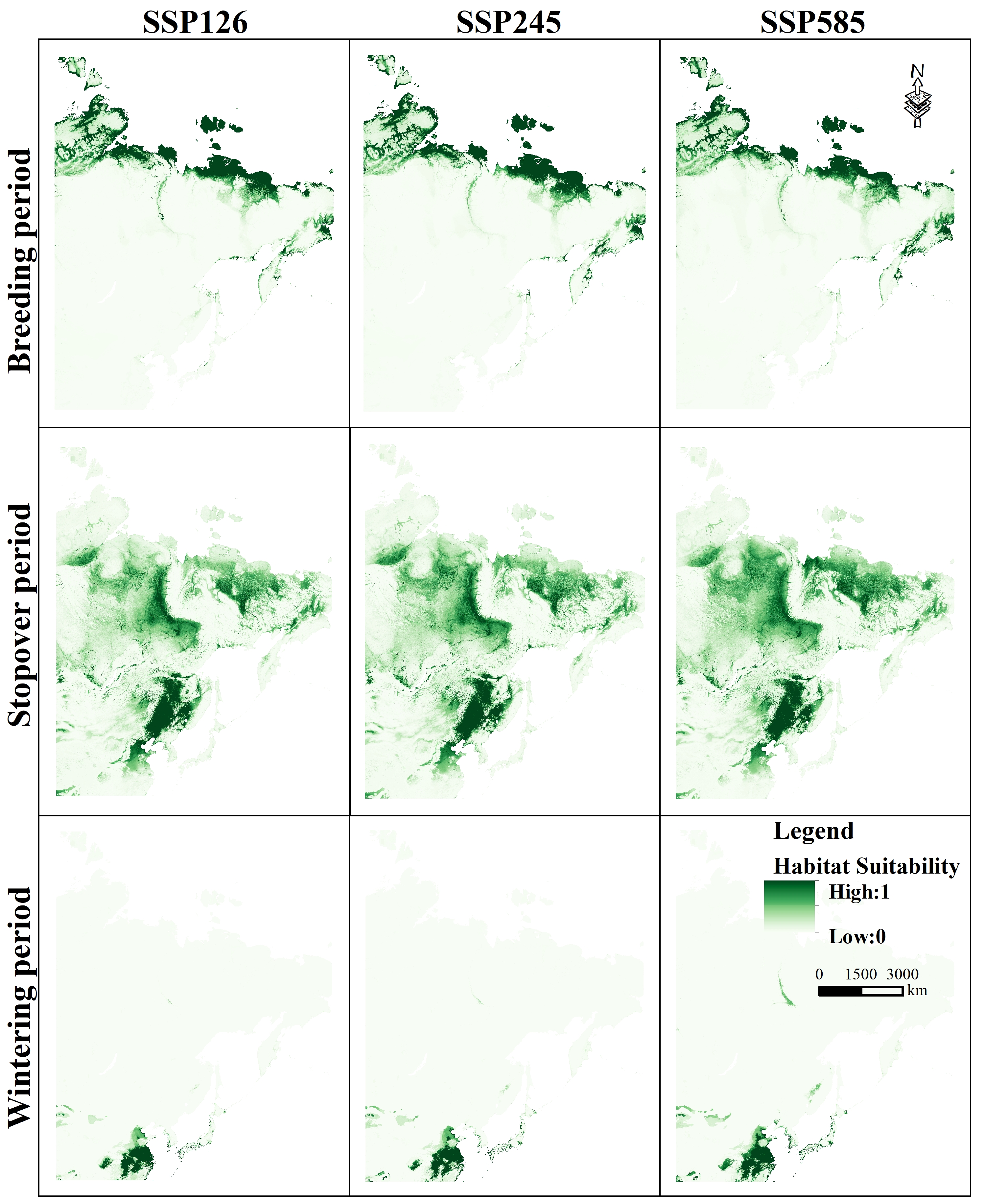


**Figure S4.** Predicted habitat distribution of GWFG under future climate scenarios.
